# The complete mitochondrial genome of *Neptunea arthritica cumingii* Crosse, (Gastropoda: Buccinidae)

**DOI:** 10.1080/23802359.2016.1155421

**Published:** 2016-03-28

**Authors:** Zhen Lin Hao, Li Meng Yang, Yao Yao Zhan, Ying Tian, Jun Xia Mao, Luo Wang, Ya Qing Chang

**Affiliations:** Key Laboratory of Mariculture & Stock Enhancement in North China’s Sea, Ministry of Agriculture, Dalian Ocean University, Dalian, Liaoning, China

**Keywords:** Buccinidae, mitochondrial genome, *Neptunea arthritica cumingii*

## Abstract

The complete mitochondrial (mt) genome of the Neptune whelk, *Neptunea arthritica cumingii*, was determined using genome walking techniques in this study. The total length of the mt genome sequence of *N. arthritica cumingii* was 15 256 bp, including 13 protein-coding genes, 21 transfer RNA genes and 2 ribosomal RNA genes. The overall composition of the mitogenome was estimated to be 30.85% for A, 38.59% for T, 15.15% for C and 15.40% for G, indicating that an A + T (69.44%)-rich feature occurs in the *N. arthritica cumingii* mitogenome. The phylogenetic relationships of 11 mollusc species were constructed based on the complete mtDNA sequences by the neighbour-joining method using MEGA 5.0 software (MEGA Inc., Englewood, NJ).

The Neptune whelk, *Neptunea arthritica cumingii*, belongs to the family Buccinidae and is delicious with internal fertilization and direct development (Richard et al. 2008). It used to be an important fishery resource in China, but stocks of this resource have been severely affected by over-exploitation and the deterioration of environmental conditions (Sui et al. 2008). Another stress factor of natural origin is the whelk’s low hatching rate, high egg mass predation rates and low juvenile survival (Sui et al. 2008). In order to restore and protect the wild *N. arthritica cumingii* resources, it is necessary to carry out wild resource investigation and germplasm analysis. In this study, we report the complete sequence of mitochondrial genome for *N. arthritica cumingii* (GenBank accession no. KU246047). The findings will provide useful information for further studies on population genetics, phylogenetic construction and other relevant studies in *N. arthritica cumingii*.

One *N. arthritica cumingii* individual was collected from Zhangzidao Island, Liaoning Province, China (39°01′92″N, 122°47′80″E). The total genomic DNA was extracted from foot muscle by a modification of standard phenol chloroform procedure. The complete mitogenome of *N. arthritica cumingii* was sequenced by primer walking. The gene annotation was performed following the methods described by Yu and Li (2012).

The total length of the hybrid of *N. arthritica cumingii* mitochondrial genome was 15 256 bp, with the base composition of 30.85% A, 38.59% T, 15.15% C and 15.40% G. It comprised two ribosomal RNA genes, 13 protein-coding genes and 21 transfer RNA genes. All the mitogenome genes were encoded on the heavy strand except for seven tRNA genes (*tRNA-Met*, *tRNA-Tyr*, *tRNA-Cys*, *tRNA-Sec*, *tRNA-Gly*, *tRNA-Glu* and *tRNA-Thr*).

The phylogenetic analysis showed that the complete mitochondrial sequence of *N. arthritica cumingii* was phylogenetically closer to *Babylonia lutosa* by 87% bootstrap support and formed the Buccinidae clade (Figure 1). Our result was consistent with the previous researches, both in traditional morphological and molecular-based phylogeny studies. We expect that the present result can contribute to construct molecular identification of this species and be helpful to explore the phylogeny of Buccinidae.

**Figure 1.**
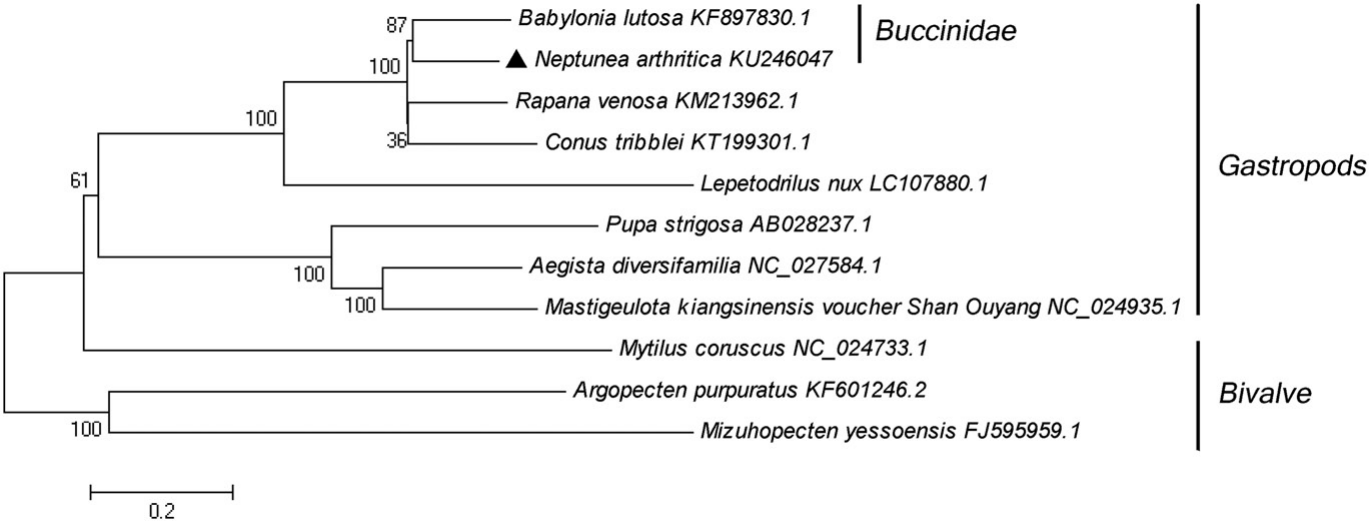
Consensus neighbour-joining tree based on the complete mitochondrial sequence of *N. arthritica cumingii* and other 10 mollusc species. The phylogenetic tree was constructed using MEGA 5.0 software (MEGA Inc., Englewood, NJ) by the neighbour-joining method. The numbers at the tree nodes indicates the percentage of bootstrapping after 1000 replicates.
